# Learning to build low-field MRIs for remote northern communities

**DOI:** 10.3389/fnimg.2024.1521517

**Published:** 2025-01-17

**Authors:** Gordon E. Sarty, Logi Vidarsson, Christopher Hansen, Keifer Corrigal, Lionel Sutherland, Millie Jamieson, Micheal Hogue, Haile Kassahun, William Greyeyes, David Teixeira, Lawrence Goertzen, Jonathan McEvoy, Mark Pollard

**Affiliations:** ^1^Space MRI Lab, quanTA Centre, University of Saskatchewan, Saskatoon, SK, Canada; ^2^LT Imaging, Inc., Toronto, ON, Canada; ^3^Aircraft Maintenance Engineering Program, Saskatchewan Indian Institute of Technology, Saskatoon, SK, Canada; ^4^Montreal Neurological Institute, McGill University, Montreal, QC, Canada

**Keywords:** accessible MRI, low-field MRI, RF encoding, STEM education, community self-determination, northern healthcare, Space MRI, MRI construction techniques

## Abstract

Low-field Magnetic Resonance Imaging (MRI) has the potential to provide autonomous accessible neuroimaging in remote communities, particularly in the Canadian north. Remoteness necessitates that these MRIs be built and maintained within the communities. This approach not only ensures that the MRIs remain operational but will also allow the youth from the communities to pursue technical careers at home. The first step in this vision is to establish that the technical resources needed for building MRIs are available in remote communities and to establish an educational program that will give students the required technical skills. Over the summer of 2024, a team of students working within an Aircraft Maintenance Engineering (AME) program built the hardware for a wrist-sized prototype MRI. The student team included a high school student, AME students, engineering students and a post doctoral fellow. The skills required to maintain aircraft, namely 3D printing, sheet metal work and electrical harness building, were sufficient to build a low-field MRI. The prototype built was a radio frequency (RF) encoding MRI, whose design was optimized for eventual use in space, but the techniques and procedures developed are applicable to other MRI designs. Furthermore the breadth of students from high school to the post doctoral fellow level facilitated an extremely rich learning environment for the students while they focused on the task of designing and building the prototype MRI. Educational programs around building low-field MRIs can be created at all levels.

## 1 Introduction

Low-field Magnetic Resonance Imaging (MRI), specifically with fields in the 10 to 100 mT range, offers a potential solution to the current lack of access to MRI experienced by most of the world's population (Geethanath and Vaughan, 2019; Webb and Obungoloch, 2023). Low-field MRI has promise for brain imaging application in remote communities with applications currently under evaluation (Kimberly et al., 2023). Potential applications include the evaluation of infant hydrocephalus (Parasuram et al., 2023; Harper et al., 2021), stroke (Yuen et al., 2022; Mazurek et al., 2023), and acute brain injury (ABI) patients on extracorporeal membrane oxygenation (ECMO) support (Cho et al., 2024).

We have been developing low-field MRI for potential use in space for astronaut health research and for medical care for future missions to the Moon and Mars (Sarty and Obenaus, 2012). To meet the stringent requirements for low Size, Weight and Power (low SWaP) for a space-worthy MRI, we have adopted a gradient-free design. Specifically, we have been developing MRIs that use the TRansmit Array Spatial Encoding (TRASE) approach (Sharp and King, 2010). Designs for Space MRIs, specifically for the *International Space Station* (*ISS*) have been developed under contract to the Canadian Space Agency (CSA) (Sarty et al., 2014). A prototype has been flown in zero-g on a Falcon 20 jet and more recently, a design for the study of the effect of interplanetary radiation on astronaut muscle and bone on the proposed *Gateway* lunar space station has been completed (Jullienne et al., 2024). The MRI prototype described in this paper is a prototype for the design for a wrist-sized MRI for the proposed lunar *Gateway* space station.

The CSA has recently announced their *Health Beyond Initiative* where they encourage the adaptation and development of medical devices and practices, originally designed for use in space, for use in northern Canadian communities. The idea is that many of the challenges faced by people living in space are also faced by people in remote northern communities. Those challenges include: *remoteness*: the healthcare facilities in large urban centers are many hours away, by aircraft for the northern community dwellers and by spacecraft for the astronauts; *limited communication*: internet and telephone service in the north may be spotty and for astronauts on their way to Mars there will be delays caused by the finite speed of light; *limited power* – power supply in the north is also spotty and power supply in space is only available from solar panels, in each case appliances need to be battery powered; *limited and expensive supplies*: in many cases, expensive air delivery is the only way supplies make it into northern communities and expensive spacecraft delivery is the only way astronauts get supplies. In both cases communities in the north and in space need to be autonomous and self-sufficient. The similarities in needs make it natural to develop low SWaP TRASE MRI designs, originally designed for use in space, for use in the Canadian north.

Learning from the lessons of deploying MRI equipment and services to remote communities in Africa (Anazodo et al., 2023), we know it is not enough to just develop and deliver the technology. In addition to remoteness from major economic centers, the need for autonomy and self-determination means that the expertise for building, maintaining and using MRIs in the community is essential. At the base for meeting that need is education. Following on that education will be the need for local entrepreneurship. Here we describe a first step in the establishment of an educational program with the successful running of a student-led summer MRI build project within the Aircraft Maintenance Engineering (AME) program at the Saskatchewan Indian Institute of Technologies (SIIT). The AME program already has a history of training students from northern communities with skills that they can take back to their communities to pursue their careers there. Aviation is central to northern community life and the AME skill-set is well suited to the construction of portable low-field MRIs.

The gradient-free TRASE MRI design described here is still in the process of development and is not quite ready for deployment in clinics. However, there are other designs that are ready and the techniques used to construct the TRASE MRI may be used to construct designs that are closer to clinical deployment. The other designs include a head-size 50 mT open-source MRI, developed at Lieden University, Netherlands (O'Reilly et al., 2021), a 70 mT extremity-sized MRI from Universitat de Valéncia, Spain (Guallart-Naval et al., 2022) and a 50 mT whole body MRI from the University of Hong Kong (Zhao et al., 2024). Other, designs that might also be built following the methods described here are an 80 mT head-size MRI (Cooley et al., 2021) and a 64 mT “MR Cap” for head imaging (McDaniel et al., 2019).

## 2 Background

After proposing to the CSA that a TRASE MRI design would be suitable for deployment on the *ISS*, we set out to build prototypes. The first MRI, which we named the *Owl MRI*, was a wrist-sized 50 mT MRI (Sarty and Vidarsson, 2018). It used a Halbach magnet design with a built-in *B*_0_ magnetic field gradient transverse to the axis of the magnet bore. This resulted in isomagnetic surfaces (natural slices) that were individually excited by varying the radio frequency (RF) transmit frequency. The approach was to use TRASE RF encoding on those surfaces to generate images. This encoding was done with a four-channel “Cube” transmit coil with four square loops. One of the four channels was run 180° out-of-phase with the other three to create orthogonal Maxwell-Helmholtz pairs for TRASE encoding (Deng et al., 2013). Images were created but signal-to-noise ratio (SNR) issues required many signal averages.

**Figure 1:**
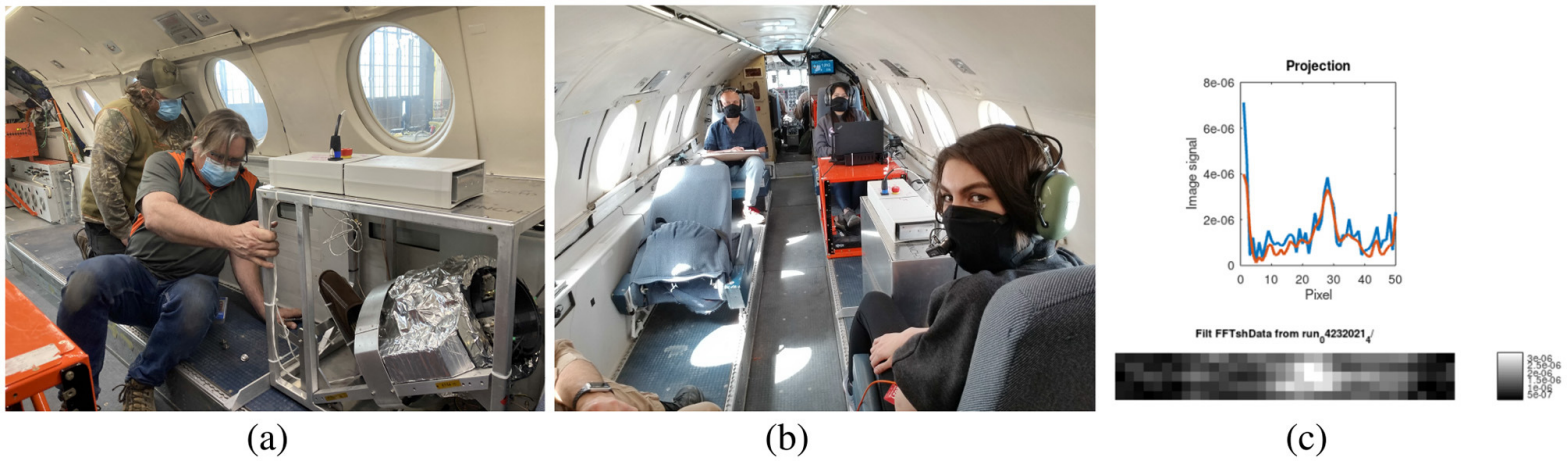
**(A)** The *Merlin MRI* being installed on the Falcon 20 jet. Here the cover is removed for installation. **(B)** Flight crew ready for zero-g. The woman in the foreground has her leg in the MRI for imaging during the 20 seconds of each zero-g parabola. **(C)** Example projection data of a volunteer's lower leg, obtained during zero-g. The projection in the top graph is from the middle natural slice. The image shows stacked projections from the corresponding axial natural slices (isomagnetic surfaces).

The next prototype built, named the *Merlin MRI*, was an ankle-size TRASE MRI made for testing in zero-g on a Falcon 20 jet. The *Merlin MRI* is illustrated in Figure 1. It had a 64 mT Halbach magnet but with an axial direction built-in *B*_0_ gradient. This defined axially oriented natural slices that would be suitable for imaging axial slices of the lower leg. The new *B*_0_ gradient direction meant that the Cube transmit coil design could not be used so the twisted solenoid coil design (Sun et al., 2019) was used, along with a saddle coil. It was intended to use two twisted solenoid coils for 2D TRASE encoding (3 independent RF phase gradients are required for 2D spatial encoding) but coupling issues (Bohidar et al., 2019) limited operation to acquiring projections. Given that only 20 seconds per zero-g parabola were available for data collection, projections were all that could be acquired in an individual parabola in any case. With the *Merlin MRI* build, the SNR problems that the previous *Owl MRI* had were resolved, enabling useful data acquisition during the available 20 seconds, plus we demonstrated that the TRASE design had the low SWaP characteristics needed for flight.

**Figure 2:**
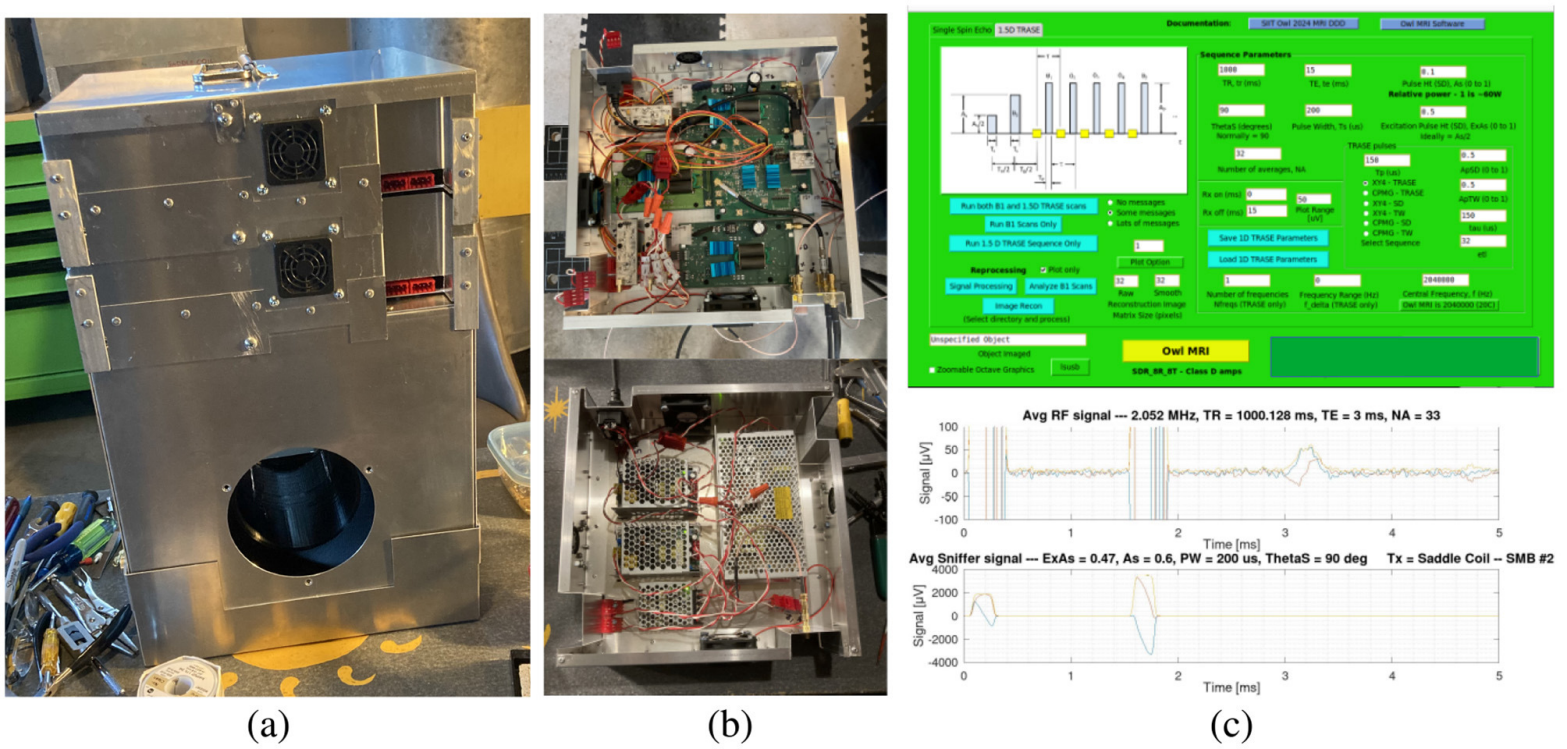
**(A)** The retrofitted *Owl MRI* of Sarty and Vidarsson (2018) referred to as the *USask Owl MRI* in the text. **(B)** The magnet is installed in the bottom box with power supplies mounted in a tray above the magnet (bottom image) and the electronics—class D RF amplifiers and an SDR (see text) are mounted in a top tray (and shown in the top image). **(C)** User interface for a TRASE sequence (top image) and an example spin echo output along with the measurement of the RF pulses via a permanently mounted sniffer loop. The *USask Owl MRI* has only produced low-resolution images to date. (A redesign of the thin saddle coil is in progress to resolve that).

**Figure 3:**
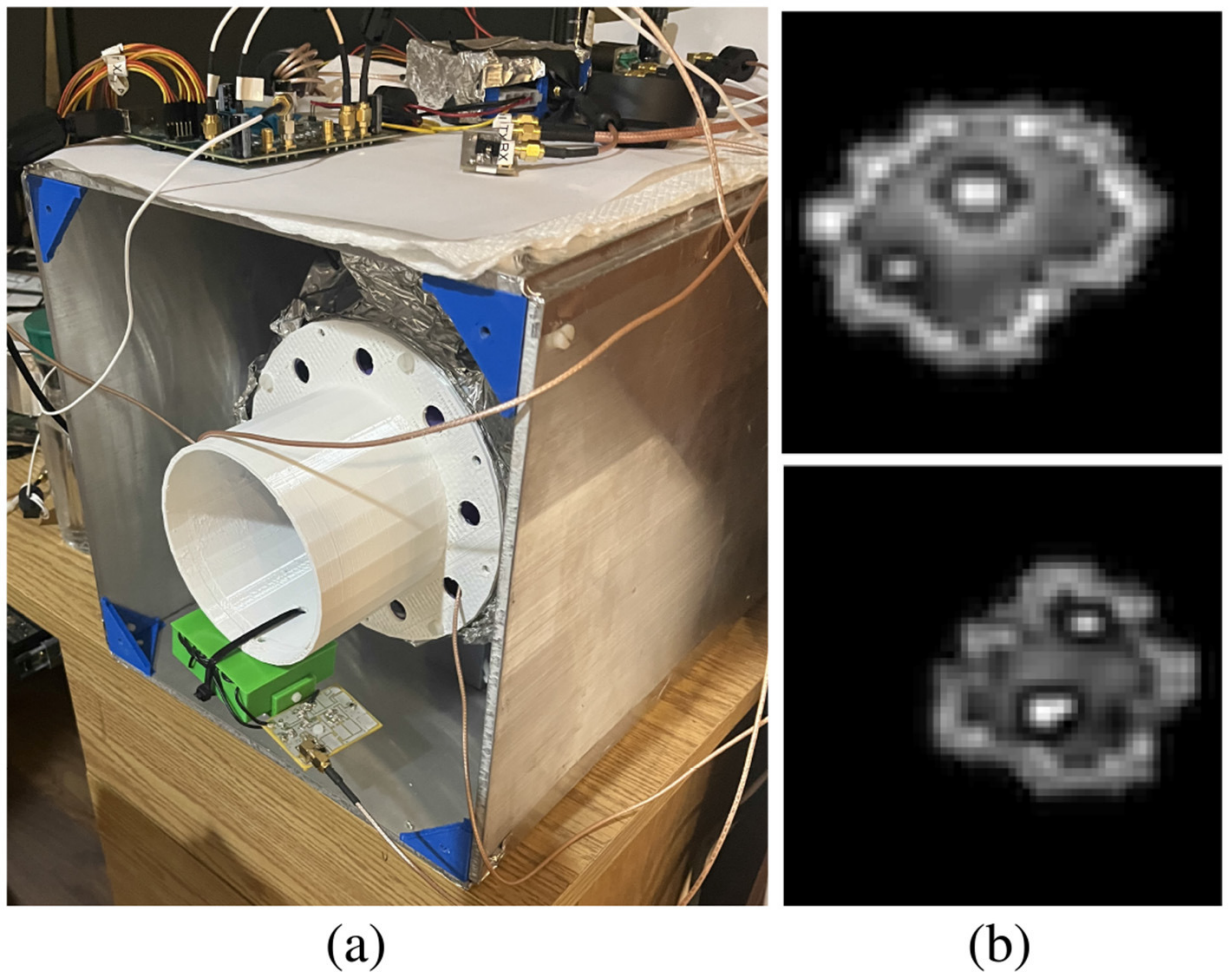
**(A)** The *LT Imaging Owl MRI*. It has very little in the way of packaging, giving ample opportunity for student-led design solutions for the *SIIT Owl MRI* build. Seen at the bottom of the picture is a test setup for the LNA/tuning board for EDITER coils (Srinivas et al., 2022) that were incorporated into the *SIIT Owl MRI*. **(B)** Wrist images made with the *LT Imaging Owl MRI* clearly show muscle, skin and the marrow and cortex of the Ulna and Radius bones. An unanticipated axial *B*_1_ phase twist in the thin saddle coil prevents further improvement of these images. Work is ongoing to redesign that coil to achieve a robust 1.5D TRASE process.

Work is ongoing with the *Merlin MRI* to uncouple the transmit coils and to implement true 2D TRASE encoding. At the same time, the original *Owl MRI* was retrofit with a twisted solenoid coil and a thin saddle coil with the objective that the thin coil produces a slice of excited spins in which the twisted solenoid-saddle coil pair provides one direction of TRASE spatial encoding and the built-in transverse *B*_0_ gradient provides the second spatial encoding direction by varying the transmit RF frequency. We call this approach 1.5D TRASE encoding (Sarty et al., 2024) where there is no coil coupling problem because the *B*_1_ directions of the two transmit coils (saddle and twisted solenoid) are orthogonal to each other. Simultaneous with the retrofit of the original Owl MRI built at the University of Saskatchewan (USask), a second Owl MRI was built by LT Imaging, Inc., a small firm in Toronto, Canada. To identify the prototypes, the original Owl retrofit we call the *USask Owl MRI* and the second, the *LT Imaging Owl MRI*. The *USask Owl MRI* is illustrated in Figure 2 and the *LT Imaging Owl MRI* is illustrated in Figure 3A. The two MRIs are both of the same 1.5D TRASE design with a difference in the Halbach magnet design. The magnet for the *USask Owl MRI* has 3 rings of magnet blocks while the *LT Imaging Owl MRI* has four rings. Operating both of these Owl MRIs revealed that the thin saddle coil had an axial *B*_1_ phase twist that prevented straight-forward TRASE spatial encoding. With some image post-processing that involved dilution, erosion, thresholding, and clamping, recognizable images could be created from data from the *LT Imaging Owl MRI* (Figure 3B). Since we had more success creating images using the LT Imaging Owl, that design was chosen for the SIIT summer student MRI build. That third Owl MRI build is named the *SIIT Owl MRI*. The methods used to construct that MRI are the focus of this work and are described in the next sections.

**Figure 4:**
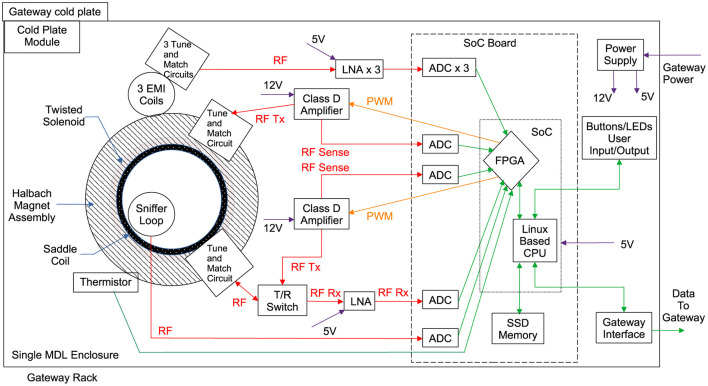
The Functional Block Diagram for the proposed *Gateway* space station MRI design. The *SIIT Owl MRI* design is based on this 1.5D TRASE MRI design. Differences between the layout shown here and the one used for the *SIIT Owl MRI* are described in the main text.

The basic design of the *SIIT Owl MRI* followed the proposed design for the *Gateway* MRI, which is summarized by the Functional Block Diagram shown in Figure 4. There are differences between the implementation of the 1.5D TRASE design for the *Gateway* and for the *SIIT Owl MRI*. The *Gateway* MRI design uses a space-qualified system-on-a-chip (SoC) board that would be far too expensive to implement for a student-built MRI. Instead, a custom Software Defined Radio (SDR) board, built by LT Imaging Inc., was used. The power supplies used, replacing power supply from the *Gateway* space station, were 5 V and 12 V switching power supplies from Mean Well Enterprises Co., Ltd. (New Taipei City, Taiwan). The low voltage needed means that these power supplies can eventually be replaced by batteries for use in remote communities. The user interface for the *Gateway* MRI was specified to be through simple buttons and light emitting diode (LED) indicators. The SDR of the *SIIT Owl MRI* is connected via a Universal Serial Bus (USB) cable to a laptop computer for the display of a graphical user interface, similar to the *USask Owl MRI* set-up (see Figure 2C). Three coils for measuring electomagnetic interference (EMI) using the EDITER scheme (Srinivas et al., 2022) are specified for the *Gateway* MRI where two were implemented for the *SIIT Owl MRI* build. Cooling for the *Gateway* MRI is to be provided by mounting the electronics on a cold plate provided on the experimental rack in the space station; natural convective cooling for the power supplies and electronics was used for the *SIIT Owl MRI* design. A thermistor for measuring the magnet temperature (because the *B*_0_ field, and therefore the operating frequency, is a function of temperature) was specified for the *Gateway* MRI but was not implemented in the *SIIT Owl MRI* built in the summer of 2024. It is intended that the *SIIT Owl MRI* be used for the development of the *Gateway* MRI design, so it is planned to add a thermistor at a future date. Other than these differences, the functional layout of the *SIIT Owl MRI* closely follows that for the *Gateway* design (Figure 4).

## 3 Methods

After some preliminary organization over the 2023–24 school year that involved testing sheet metal working techniques and the acquisition of 3D printing equipment, a team of summer students was hired to work on the MRI build project. The team was a unique mix of students from high school level to the post doctoral level. In particular, the core of the team was composed of engineering (mechanical and engineering physics) students and AME students. A large part of the activity—the educational experience—involved cross education between the students. The AME students worked with and taught engineering students how to do sheet metal work (cutting, bending, and riveting) and parts were made by all students. The engineering students taught the AME students how to use Computer Aided Design (CAD) software and all students were involved in the design of the packaging for the MRI. The AME students and the engineering students worked together to assemble a new 3D printer, optimize the print settings and to print the magnet structure, RF coil parts and other small parts as needed for the MRI (including a set of plastic screwdrivers and wrenches for working around the magnet).

The student team leader was trained as an AME at SIIT, worked as an AME for a number of years and returned to university for training as a mechanical engineer. The team leader was essentially the project manager and assembled a Deliverable Tree and Gantt Chart in the first few days of the project. The project plan was followed, and completed, over the months May to August, 2024, and a completed *SIIT Owl MRI* was delivered. In addition to the physical MRI, a series of technical and pedagogical manuals were also delivered. To give some visibility into the organization of the team, we list the members here by role, along with the period they worked on the project if it was not the entire four months:

1. Student team leader/Project manager. Trained AME and 4th year USask Mechanical Engineering student.
2. Two 1^*st*^ year SIIT AME students.
3. One 3^*rd*^ year USask Mechanical Engineering student.
4. One Engineering Physics graduate who started an MSc program following the summer project.
5. One visiting postdoctoral fellow from McGill University, getting ideas for similar MRI build projects in Africa and working on a redesign of the saddle coil. Joined the team for the month of May.
6. One grade 11 high school student. Joined the last week of June and worked until the end of August.

The documentation produced is open source and is posted to the link given in the Data Availability Statement. Not included in the documentation is any information about the Halbach magnet itself. The magnet design is protected by a patent (Vidarsson, 2018) owned by LT Imaging, Inc. and licensing arrangements are required to build that design.

## 4 Results

**Figure 5:**
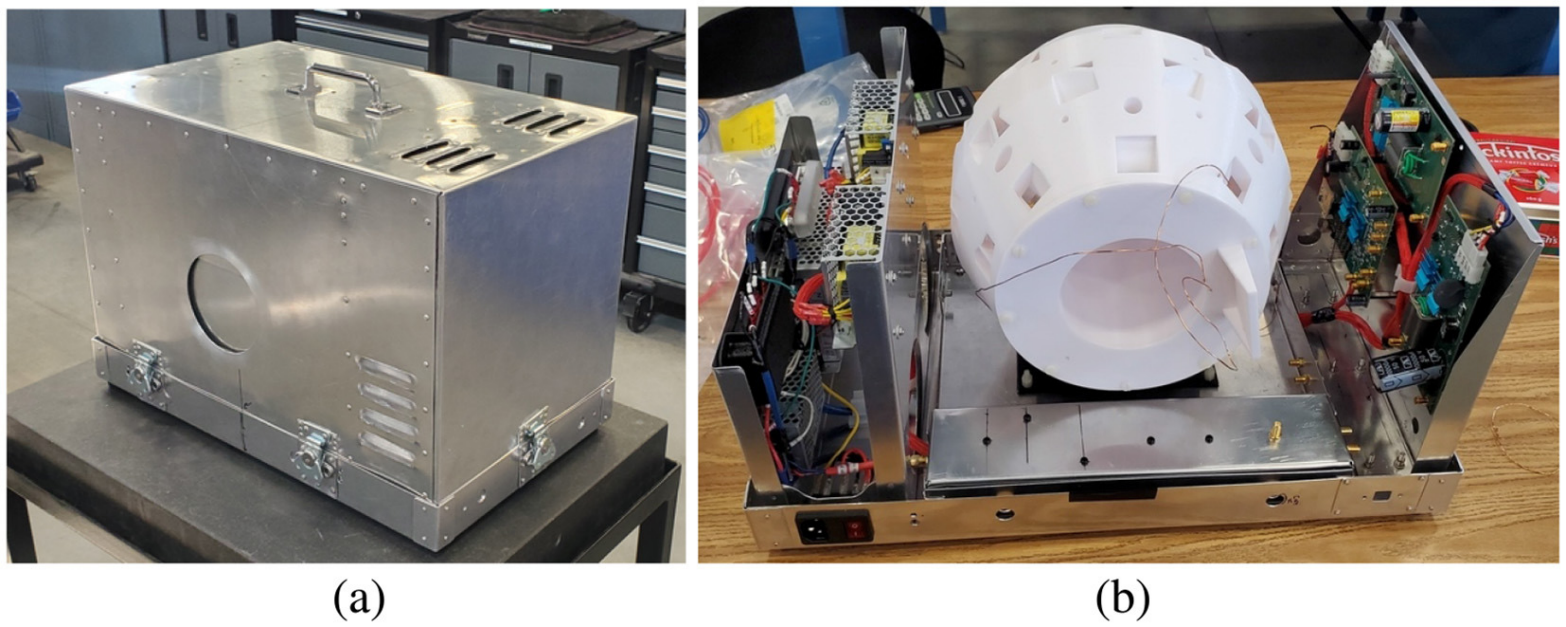
The *SIIT Owl MRI* that was built by the student team during the summer of 2024. **(A)** The completed MRI package. **(B)** A view of the MRI, with the covers removed and before the coils were finished and the magnet blocks installed.

A physical *SIIT Owl MRI* prototype was created, see Figure 5, along with pedagogical and technical documentation describing how the MRI was built and how to build it. The documentation produced was:

- Illustrated Parts Catalog.
- Wiring and Electrical Manual.
- Technical Drawing Package.
- General 3D Printing Guide.
- Coil Assembly Manual.
- Manufacturing Skill Guide.
- Document Control Procedures.

A description of the documentation follows.

### 4.1 Illustrated Parts Catalog

**Figure 6:**
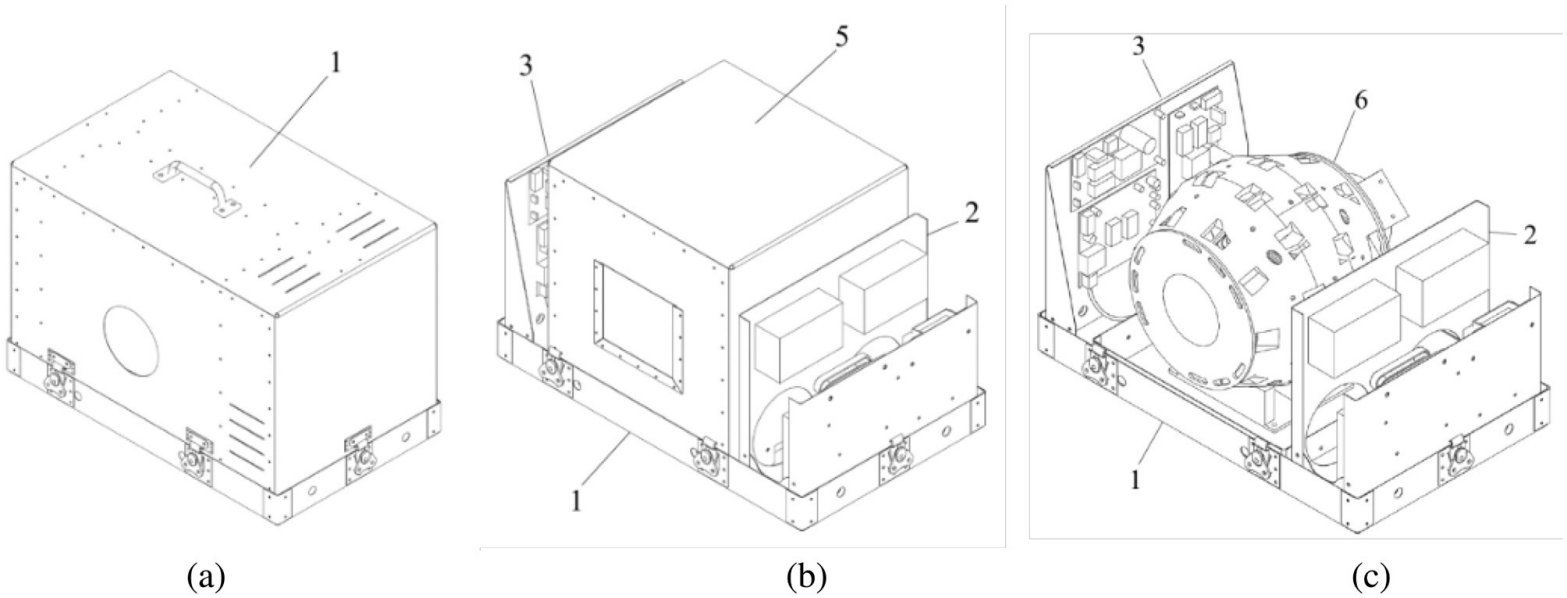
The top level assemblies for the *SIIT Owl MRI* as given in the *Illustrated Parts Catalog*. **(A)** The complete assembly, ready for use. 1 labels the Outer Shell - Top Cover. **(B)** With the top cover removed, 1 labels the Outer Shell - Tray, 2 labels the Power Mount on which the power supplies are mounted, 3 labels the TR Mount on which the two class D amplifiers and the SDR are mounted and 5 labels the Isolation Chamber - Top Cover. **(C)** With the isolation chamber cover removed, the Magnet, 6, is visible. In the *Illustrated Parts Catalog* the numbers actually refer to subassemblies as follows: 1, Outer Shell Installation; 2, Power Supply Installation; 3, Transmit/Receive Installation; 4, LNA Installation; 5, Barrel Chamber Installation; 6, Magnetic Barrel Installation. The LNA is not visible in the views shown here.

The *Illustrated Parts Catalog* was organized the way a parts catalog for an airplane would be organized. AMEs become familiar with such documents in their training and consult with them as they disassemble, repair and reassemble aircraft subassemblies. The document is mainly illustrations showing the subassemblies in increasing detail. The top level assemblies, from the document, are shown in Figure 6.

### 4.2 Wiring and Electrical Manual

The following parts, designed and built by LT Imaging Inc., were supplied to the MRI build team:

- Software Defined Radio (SDR). This custom piece of hardware primarily consists of a Field Programmable Gate Array (FPGA) that runs the RF pulse sequences through the use of digital Pulse Width Modulated (PWM) transmit output for the class D amplifiers and receives the MRI signal from the Low Noise Amplifier (LNA) through Analog-to-Digital Converters (ADC–Transceivers). It is connected to a laptop via a USB cable and requires a 5V DC power supply.
- Class D amplifiers. Two Class D amplifiers are required for the 1.5D TRASE configuration. The use of Class D amplifiers over the more traditional Class A/B amplifiers saves considerable weight and power. The Class D amplifiers require 12V DC power.
- T/R Switch. A passive Transmit/Receive switch is used on the saddle coil.
- Low Noise Amplifier (LNA). Placed in the receive chain between the T/R switch and the SDR. It was housed in it's own sheet metal box to shield it from noise. The LNA requires 12 V DC power.

The *Wiring and Electrical Manual* details how to make custom SMA co-axial cabling for the MRI along with the other cabling for power supply. The cabling was laid out on a harnessing board similar to how cabling for an aircraft gauge panel is laid out. All wires were labeled for error-free assembly. The *E-3-001 - 2024 SIIT Owl MRI Wiring Schematic (A4)* drawing is an integral part of the wiring procedure described in the *Wiring and Electrical Manual* and shows a schematic of the complete *SIIT Owl MRI* wiring harness detailing how to connect the electronics boards listed above to each other and to the RF coils. The electronics boards themselves are not open source designs and are available from LT Imaging, Inc.

### 4.3 Technical Drawing Package

In addition to the documents proper, drawings were made using the Fusion 360 software. The drawings are part of the Technical Drawing Package. In order to be open access, the drawings made with the Fusion 360 software were saved in step format. The step format files may be opened with FreeCAD, for example. As part of the documentation, all drawings have been posted to the link given in the Data Availability Statement. The drawings produced are listed in Table 1.

**Table 1:** Technical drawing package.

**Main assemblies**	**Miscellaneous assemblies**
1. Outer shell	– Various coils
– Outer Shell - Bumpers.step	– EDITER_Coil_Former_V2.step
– Outer Shell - Butterfly Latches.step	– SaddleCoil.step
– Outer Shell - Latch Doublers.step	– SaddleCoilHandle.step
– Outer Shell - Top Cover.step	– SinePhiCoil.step
– Outer Shell - Tray and angles.step	– SinePhiHandle.step
2. Power Mount	– TwistedSolenoidCap.step
– Bus Bar 1.step	– TwistedSolenoidCoil.step
– Bus Bar 1 Shield.step	– Various Subassemblies
– Bus Bar 2.step	– 1-8 inch screw.step
– Bus Bar 2 Shield.step	– 6024T16_SMA Connector Junction.step
– Power Mount.step	– 7565K46_Nylon Cable Holders.step
– Power Switch.step	– Black Nut.step
– Power Switch Shim.step	– Black screw.step
– PSU1-RS-100-5.step	– Flat rivet.step
– PSU2-RS-25-5.step	– Hex Standoff.step
– PSU3-4-RPS-200-12-C.step	– Rivnut Assembly.step
3. TR Mount	– Universal Rivet.step
– Class D Amplifier V 3.0.step	– All Sheet Metal.step
– Data Port.step	– PLA Tool Files
– SDR 8R 8T V1.2.step	– 4mm Socket.step
– TR Mount.step	– 4mm Wrench.step
4. LNA	– 6mm Socket.step
– LNA 2.08 MHz.step	– 7mm Socket.step
– LNA - Tray and angles.step	– Long Flathead Screwdriver.step
– TR Switch.step	– Short Flathead Screwdriver.step
5. Isolation Chamber	– Stubby Flathead Screwdriver.step
– Isolation Chamber - Top Cover.step	**Engineering Drawings**
– Isolation Chamber - Tray.step	– Deliverable Tree.pdf
– Isolation Chamber - Vibration-Damping Screws.step	– Gantt Chart.pdf
6. Magnetic Barrel	– E-3-001 - 2024 SIIT Owl MRI
– Editor Coil.step	Wiring Schematic (A4).pdf
– Magnet.step	– Barrel Cover Drawing v1.pdf
– Magnetic Barrel - Barrel Base.step	– MRI Tray Drawing v2.pdf
– Magnet Shims.step	– Power Mount Drawings v2.pdf
– Nylon Hex Nut.step	– Top Cover Drawings v7
– Nylon Screw.step	– T_R Mount Drawing v2
– Saddle PCB.step	
– Sniffer loop.step	
– Sniffer Loop Shim.step	
– Twisted Solenoid PCB.step	

### 4.4 General 3D Printing Guide

The *General 3D Printing Guide* is a pedagogical manual produced for use in future SIIT, and potentially high school industrial arts class offerings. The manual focuses on the 3D printers available at the SIIT, namely the Tronxy VEHO 600 Pro printer (Shenzhen Tronxy Technology Co., Ltd, Shenzhen, Guangdong, China) and a locally made CR-10 Wave printer (Wave of the Future 3D Printing, Saskatoon, Saskatchewan, Canada). For wider use, the Guide may be adapted for other printers.

### 4.5 Coil Assembly Manual

A very detailed guide is given to the process of making the following RF coils for the *SIIT Owl MRI*:

- Sine-phi coil—Given the axial *B*_1_ phase twist associated with the original thin saddle coil design, a longer “sine-phi” coil was made to take its place for preliminary testing. It will not produce the thin slice that is part of the 1.5D TRASE approach but will allow the imaging of tubes until the thin slice coil design is perfected. The coil has wire spacings matched with the theoretical sine variation of a 2D current density on a cylindrical surface needed to produce a uniform *B*_1_ field inside the coil. The construction of the tune and match circuit for the coil is also described.
- EDITER coils—Two coils meant for the detection of EMI while imaging were made. The setup of the dedicated LNA board for the EDITER coils is described in addition to the 3D printing process.
- Twisted solenoid coils—The construction of this specifically TRASE RF coil is detailed along with the procedures for making the tune and match circuit.

**Figure 7:**
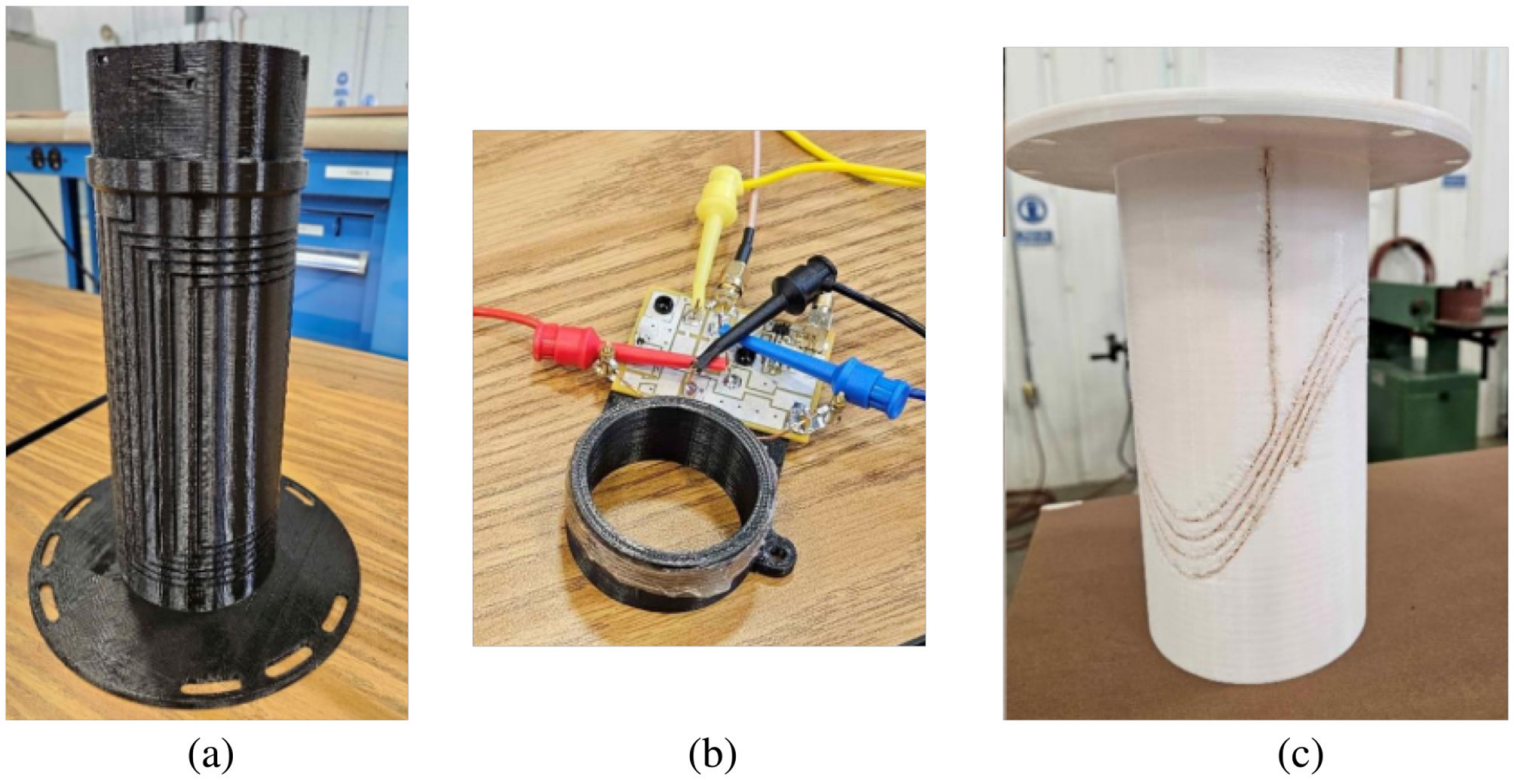
Images from the *Coil Assembly Manual*. **(A)** The sine-phi coil (temporarily standing in for the thin saddle coil). **(B)** An EDITER coil being tuned. **(C)** The twisted solenoid coil.

The requirement for nested RF coils meant that the coils were made thin, radially, to minimize the space taken inside the magnet bore. This required printing grooves so that the wire wound into the grooves would be flush with the coil cylinder surface. That way the sine-phi (or a thin saddle) coil can be rotated within the twisted solenoid coil to achieve inductive decoupling between the two coils. The groove depth specified in the CAD drawings does not exactly match the depth finally printed because of sagging of the heated plastic while printing. Therefore, this document also gives the detailed procedure used to empirically determine the groove depth to use in the CAD drawings to achieve the desired groove depth in the final printed part. Images of constructed coils, taken from the *Coil Assembly Manual*, are shown in Figure 7.

### 4.6 Manufacturing Skill Guide

The *Manufacturing Skill Guide* is another pedagogical document teaching the skills required for sheet metal working. This manual will be used to establish a curriculum for MRI-build-centered industrial-arts classes for high schools in northern Saskatchewan communities. It will also be used to train new groups of SIIT summer student teams for future summer MRI building projects.

### 4.7 Document Control Procedures

The student team took a very systematic and disciplined approach to the development and writing of the documentation for the summer MRI build project. This final document outlines the process used for the team writing exercise that was needed to produce the documentation. The procedures will be used for future MRI build projects.

## 5 Discussion

In order to effectively use a medical imaging modality like MRI in a remote community clinic, the community needs to be familiar with, and understand, the technology. Past experience points to the vision that the MRI should be built and maintained in the community. This approach has several advantages that can make the difference between the effective use of the MRI on a day-to-day basis and seeing the MRI gather dust in a corner of a clinic because it is broken and/or no one knows how to use it.

The best way to establish a highly educated work force in a small community is to educate the youth in that community. Living conditions in northern Canadian communities are harsh, especially in winter, and the retention of highly educated workers, like nurses and teachers, brought in from the outside is problematic. The solution is to educate the people who already live there and who want to stay because that is where their family is. With aviation being a central part of life in northern Saskatchewan communities, the SIIT has had success in educating people in the art of repairing aircraft, through the AME program, with the students being able to find work in their home communities at the conclusion of their training. Our aim is that low-field MRI manufacturing can be done following a similar model. We have shown that the educational resources already available through the SIIT's AME program are enough to train people how to build low-field MRIs. Of course, for aircraft maintenance, the economic infrastructure already exists to supply jobs for AME graduates. Airlines that service the northern communities need mechanics in the communities and they can hire the AME graduates. Manufacturing MRIs in northern communities will require an entrepreneurial effort on the part of the community members. But such an effort can have a long term stable effect on a community that is not available from, for example, large mining companies that traditionally hire people from the communities. A local entrepreneurial effort will benefit the community directly as opposed to benefiting a large company not based in the community. When a mine closes, the jobs disappear and there is little that a community can do about it. A community based manufacturing business will give the community more autonomy around the economy built around such a business. We believe that the development and dissemination of open source material on how to build MRIs is necessary to reduce start-up costs and reduce the risk for future community-based entrepreneurs. The material developed over the summer MRI build project has been made open source and complements open source material that already exists on the Open Source Imaging website at https://www.opensourceimaging.org/.

Apart from economic angles, it is well-known that an educated community is needed to make MRI an effective and viable option for a community (Anazodo et al., 2023). Participating in a low-field MRI building project is a very effective way to learn about MRI technology, especially for repair and maintenance technicians. Basic knowledge learned working with low-field MRIs will be applicable to high field MRIs, where it is possible to install such MRIs in the community. Workshops based around the building techniques demonstrated here would be applicable to other low-field MRI designs, particularly the Leiden-designed open source MRI that groups around the world are now building (Obungoloch et al., 2023).

The TRASE MRI designs are optimized for use by astronauts in space in terms of SWaP. The wrist-size MRIs (the Owl MRIs) have masses around 12 kg. The ankle size MRI (the Merlin MRI) has a mass of around 39 kg. Further design optimization can reduce these masses. The Owl MRI designs consume an average of around 30W, with peaks of around 150W, while imaging. The Merlin MRI uses approximately 3 times more power. The masses of the Owl MRI magnet assemblies are approximately 5 kg and the mass of the Merlin magnet assembly is approximately 15 kg. The power supplies for the Owl MRIs have a combined mass of approximately 5 kg leaving about 2 kg for the electronics and structure. In particular, the Class D RF amplifiers are extremely light and small compared to the Class A/B RF amplifiers normally used for MRI. These conventional amplifiers typically have a mass of 5 kg each and occupy a rack size volume (roughly 480 mm × 480 mm × 45 mm). Gradient amplifiers have a similar mass and volume footprint. So a conventional (non-TRASE) MRI will need at least four rack size volumes (three gradient amplifiers are needed) with a total mass of roughly 20 kg. That amplifier package will need to be installed in a small rack separate from the magnet assembly. All of the electronics, and power supplies, are mounted in the same box as the magnet with the *SIIT Owl MRI* build.

The *SIIT Owl MRI* is a wrist-sized prototype. Plans are to build another wrist-sized prototype in the summer of 2025 that more faithfully represents the severe space constraints for scientific instrumentation on the lunar *Gateway* space station. For the summer of 2026, a head-sized MRI is currently planned. Such an MRI will, of course, then have neurological applications. The utility of low-field MRIs is currently being debated and investigated by many researchers. They certainly cannot replace the high field MRIs that now exist only in large urban centers. Travel from remote communities will still be required for many conditions that now benefit from an MRI exam. So from that perspective, the impact of low-field MRI on the wider problem of MRI accessibility may be limited, especially of MRIs with fields in the 10 to 100 mT range. However, our hope is that low-field MRIs will be seen as a somewhat new medical imaging modality with applications not yet currently imagined. For example, an easily accessible low-field MRI might be used to assess the severity of frost-bite—a condition of more importance in northern communities than in large urban centers—and is a condition for which one would not dream of using an expensive high field MRI for diagnosis.

We have completed an essential component of the education step needed to realize the objective of building MRIs directly in community medical clinics and have discussed the possible clinical uses that the low-field MRIs might have. A third component needed before this self-contained model of diagnostic MRI service can be realized is that of device certification by the relevant authority, in our case by Health Canada. No work has been done yet toward establishing a process for certification. Ideally an open source design would be certified given that it is built according to detailed build instructions similar to the documentation written by the students for the *SIIT Owl MRI*.

## 6 Conclusion

Low-field MRI, defined here as MRI with *B*_0_ in the 10 to 100 mT range, has the potential to make neuroimaging available in remote communities. Neuroimaging data collected in communities may be transmitted to larger centers for assessment by specialists. Low SWaP designs such as gradient-free TRASE MRIs currently designed for eventual use in space, especially on and around the Moon, offer an optimal solution for remote communities. This is especially true for northern communities where access is frequently only via aircraft. Sustainable use of MRI in remote communities will only be possible if the MRIs can be easily built and maintained autonomously in the community. We have shown that the techniques and skills needed for aircraft repair, an activity that is routine in northern communities, may be used to construct low-field MRIs. This has been demonstrated for a particular type of gradient-free MRI design, but the technique can be applied to other open source MRI designs now available. Building MRIs also provides an effective educational focus that could be applied to high school classes as well as to trade school classes. The target educational goals are to simultaneously learn about how MRIs work and to learn the manufacturing skills need to make the MRIs. Over the course of four months in the summer of 2024, a team of students working with the AME program at SIIT demonstrated that a complete packaging design and build of a given MRI design is feasible in that timeframe—the time frame of one school term in high school, trade school or university. Technical and pedagogical documentation was created that other groups can use to build up either start-up businesses for building low-field MRIs or for developing similar STEM-oriented educational programs.

## Data Availability

The datasets presented in this study can be found in online repositories. The names of the repository/repositories and accession number(s) can be found below: https://git.cs.usask.ca/ges125/2024-siit-mri.
